# Comparative evaluation of nano ocular delivery systems loaded pH and thermosensitive in situ gels for Acanthamoeba keratitis treatment

**DOI:** 10.1038/s41598-025-03418-5

**Published:** 2025-06-03

**Authors:** Basant A. Abou-Taleb, Ibrahim A. Abdelwahab

**Affiliations:** 1 Department of Pharmaceutics and Pharmaceutical Technology, Faculty of Pharmacy, Pharos University in Alexandria, Canal El Mahmoudia Street, Beside, Green Plaza Complex, Alexandria, 21648 Egypt; 2 Department of Pharmaceutics and Pharmacy Practices, Alexandria University Hospitals, Alexandria University, Alexandria, Egypt; 3 Department of Microbiology and Immunology, Faculty of Pharmacy, Pharos University in Alexandria, Canal El Mahmoudia Street, Beside, Green Plaza Complex, Alexandria, 21648 Egypt

**Keywords:** Propamidine isethionate, *Acanthamoeba keratitis*, In-situ gel, Nano-ocular systems, Chitosan/Pluronic F-127, Microbiology, Diseases, Nanoscience and technology

## Abstract

*Acanthamoeba keratitis* is the most dangerous ocular-infection that can cause blindness. The propamidine-isethionate (PI) 0.1% eye-drops are used in the treatment, but they have low-bioavailability due to precorneal loss parameters. In-situ-gels are administered as drops into the eye and changed into gel in the cul-de-sac, which may be able to solve these issues. In this study, we demonstrate different nano-ocular delivery-systems formulations of 0.1%PI loaded in-situ-gels using the ideas of pH & temperature-stimulated in-situ-gelation. Pluronic F-127 a thermosensitive-polymer with chitosan as a pH-sensitive polymer that also enhances permeability was utilized as a gelling-agent. Several *in-vitro* pharmaceutical and antiprotozoal assessments on *Acanthamoeba keratitis *were assessed for the developed formulations. It was observed that the created PI-chitosan nanoparticles in-situ-gel formulation showed smaller particle-size, higher zeta with a higher %inhibition of amoebae after 24-h incubation compared to other formulations with continuous drug release for 24-h. The PI-CSNPs in-situ-gel formulation offers an effective substitute for traditional BROLENE eye-drops in the management of *Acanthamebae*, around double the effect based on % amebae inhibition, reached 92%. It can reach the clinical-stage & is appropriate for sustained-ocular-administration for the treatment of Acanthamoeba keratitis.

**Supplementary Information:**

The online version contains supplementary material available at 10.1038/s41598-025-03418-5.

## Introduction

Acanthamoeba keratitis (AK), a severe sight-threatening corneal infection, has become a significant medical problem, especially among contact lens wearers^1^. The disease manifests as eye pain, congestion, blurred vision, lachrymation, and ring-shaped infiltrates of the cornea, and can lead to permanent blindness. The amoebae are commonly detected in water, including swimming pools and air conditioners, as well as in soil and dust. More than 90% of AK cases are diagnosed in the contact lens user population. Contamination of contact lenses with amoebae during storage or while wearing may be the first step in AK development. Popular contact lens solutions mainly include topical anti-fungal and anti-microbial ingredients and are not fully efficient against Acanthamoeba trophozoites and cysts^2–4^. Wearing contact lenses may cause micro-corneal damage and ulcerations, which act as anchor points for Acanthamoeba adhesion. The amoebae can simply transfer from the contact lens surface to corneal epithelial cells^5^. AK infection affects corneal stroma and finally leads to nerve infiltration. The advanced stage of AK manifests itself as eye pain, lacrimation, redness, eye congestion, blurred vision, and a foreign-body sensation^6^. When *Acanthamoeba keratitis* worsens and the infection is incurable, the damaged eye has to be removed^7^. Propamidine isethionate (0.1%) eye drops are a traditional ocular dosage form used in the treatment of AK^8^.

Although the traditional ocular drops are the most simple type of drug delivery system (DDS), they have several disadvantages, including the need for frequent dosing, dilution, & drainage of the drug by tear fluid, decreased bioavailability, etc.^9^. Novel ocular drug delivery systems, which use viscosity/penetration enhancers, gel-like substances, colloidal DDS, nanoparticles (NPs), inserts, in-situ developing gels, O/W emulsion, lipid nano-systems etc., have been created to extend the duration of contact to solve the disadvantages of the traditional dose forms^10,11^.

Polymer solutions known as "in-situ gels" can be administered in a liquid state and, when exposed to physiological conditions, change into a semisolid gel. Changes in pH, temperature, and the presence of ions may all trigger the gelation to occur^12^. They are easily administered into the eye’s conjunctival sac as a solution. The system transforms into a clear, translucent gel as it comes into contact with the eye. The advantage of this kind of formulation is that the gel’s favorable residence time makes the solution patient-convenient. Therefore, in-situ gels that can increase bioavailability, decrease the frequency of administration, lengthen the drug’s corneal contact duration, and be patient-friendly are required.

Three-dimensional (3D) cross-linked network structures called in-situ hydrogels can swell significantly when a lot of water is added^13^. Among them, thermosensitive in-situ hydrogels which are useful for drug delivery in biomedicine. Thermosensitive, also known as poloxamers, have been thoroughly studied as gel-forming substances in-situ^14–18^. Poloxymers (Pluronics), are commonly employed as non-ionic surfactants as well as solubilizers^19^. They have been studied for use in oral, rectal, ocular, nasal, and vaginal applications and have been recognized as safe (GRAS) excipients^20^. The poloxamer 407 (P407), also known as Pluronic F-127, has been thoroughly investigated for usage in the ophthalmic products and has the lowest toxicity and lowest critical gelation concentration of all the poloxamers^19^. F-127 also possesses wound-healing properties^21^. During periods of high shear stress, such as while the eye is blinking, poloxamer in-situ gel displays pseudoplastic shear thinning flow, which results in uniform spreading above the corneal surface while remaining kept on the surface^22^. In solution, they create micelles that, based on the temperature and concentration of the polymer, can self-organize & form a viscous gel^23^. Despite being widely used, thermosensitive copolymers have a significant disadvantage in that their limited mechanical strength causes quick degradation^24^. Using mixes of poloxamers and chitosan to solve this issue is an innovative strategy^25–27^.

Chitosan is a linear polysaccharide that is pH-dependent, biodegradable, biocompatible with the eyes, has anti-inflammatory and antimicrobial effect^28–30^which dissolves in water up to a pH of 6.2. For ocular applications, chitosan is regarded as the preferred cationic polymer; It has many free amino groups that give it a positive charge, which it may utilize for interaction with the negatively (-ve) charged corneal surface mucin^31,32^. There is evidence that chitosan may improve the absorption of drug molecules into the cornea^33,34^. Its mucoadhesive capabilities are thought to function as a penetration enhancer. It was viscous, bioadhesive, and capable of gelling at ocular pH 7.4^35^. Gels developed by neutralizing chitosan with polyol salts have pH-sensitive gelling characteristics^36^. When a chitosan solution is neutralised with a weak base, the system remains in solution at ambient temperature & physiological pH, but it will gel when heated to physiological temperature (37 °C)^35^.

Nanotechnology is a valuable science that includes the development & use of novel controlled Nano-metric size particles for the management of various ocular illnesses^37^. Several advantageous characteristics of nanoparticle (NP)-based delivery systems have made them desirable options for developing ocular applications^38^. Among all the nanocarriers, polymer-based nanocarriers (such as chitosan NPs) and lipid-based nanocarriers (such as NLC and Liposome) are most promising for ocular drug delivery^39^. In the past decade, chitosan-based nanoparticulate systems have garnered significant attention for addressing and overcoming the limitations of conventional ocular pharmaceutical products, including inadequate residence time and low corneal permeability^40^. The chitosan (CS) nanoparticles provide several advantages such as non-toxic, biocompatible, bioactive, being able to form stable, appropriately sized NPs with ease and without causing irritation; and having a mucoadhesion effect because of electrostatic interaction and hydrogen bond formation between the cationic polymer and the anionic ocular mucin^41^. These factors also improve the permeability by relaxing the tight connections between cells and increase the residence time of drugs and their ocular bioavailability^40,42,43^. Chitosan also possessed antifungal, antibacterial, and wound-healing properties. According to these unique qualities of CS (such as mucoadhesion to the cornea, biodegradability, antibacterial qualities, etc.^44^) make it an appropriate nanomaterial for the development of an ocular drug delivery nanomedicine in the treatment of AK and they are excellent polymeric-based nanocarriers choices for ocular applications^40^. A number of lipid-based nanocarriers (LPN), including liposomes, solid lipid nanoparticles (SLN), nanostructured lipid carriers (NLC), etc., have also been used in ocular nanomedicine research in recent years, with the goal of improving the ocular bioavailability of various drugs and biologics because of their good solubility, drug loading, and target specificity for the advancement of ocular therapeutics^45^. NLC formulations have been chosen over SLN for ocular delivery because they can penetrate the ocular epithelial layer with greater efficiency and stay on the cornea for longer residence time^46,47^. Additionally, NLCs can provide a number of benefits for ocular administration, including good corneal residence duration, regulated and sustained drug release, high tolerability, and lipid biodegradability (Generally Recognized As Safe; GRAS)^47,48^. Liposomes have been investigated for ocular-drug-delivery since it offers advantages as a carrier system. It is a biodegradable & biocompatible nanocarrier. They can enhance the drug-permeation by binding to the corneal-surface & improving residence period^49^. They were widely employed for ocular medication delivery due to their excellent corneal penetration capacity and affinity via epithelial cell membranes^50^. It can encapsulate both the hydrophilic & hydrophobic drug molecules. Furthermore, liposomes can improve pharmacokinetic profile, enhance therapeutic effect, and minimize toxicity associated with higher dose. Liposomes have been widely investigated for the treatment of both anterior and posterior segment eye disorders due to their versatility^49^. They showed promising results in ophthalmic-drug-delivery and have significant clinical application potential in the treatment of ocular disorders^51^.

The current work chose to formulate in-situ gels using a chitosan/Pluronic F-127 combination as a vehicle for improved corneal permeability, better corneal mucoadhesion, and prolonged release of propamidine isethionate (PI) for the treatment of AK. The aim of this study was to formulate a pH-thermosensitive, mucoadhesive, in-situ gel for ophthalmic use that contained PI encapsulated in various nanosystems, including liposomes, nanostructured lipid carriers (NLC), and chitosan nanoparticles. The formulations were then characterized *in-vitro* in order to evaluate its pharmaceutical and antiprotozoal characteristics. A commercial Propamidine isethionate 0.1% Eye drops (BROLENE Eye Drops, Sanofi Aventis, Egypt) was used as a source of comparison in this study which is frequently recommended for treatment AK by physician.

## Materials and methods

### Materials

#### *Acanthamoeba keratitis* isolation and Trophozoite cultivation

Fifty (50) contaminated contact lens solutions were taken from the I-Care Hospital and El Safwa Eye Center laboratories during the period from 8–2023 to 11–2023. Solutions were vortexed, and 0.5 mL of lens solution was inoculated over the surface of a non-nutrient agar plate seeded with a dense suspension of Escherichia coli (NNA-E.coli). Using a light microscope, the incubated Non-nutrient agar plates were screened daily for signs of Acanthamoeba growth for 7 days. After 3 days of incubation, the NN agar showed various shapes of trophozoites with acanthopodia. Isolates of free living Acanthamoeba (FLA) were identified by morphologic examination of the trophozoite and cyst forms^52,53^. Trophozoites were washed twice in phosphate-buffered saline (PBS) buffer (pH 7.4). Trophozoites were concentrated by centrifugation at 500 g for 10 min. Trophozoites were counted in a hemocytometer, adjusted to a final concentration in PBS at a density of 8 × 10^5^ amebae/mL (95% trophozoites), and used immediately for testing^3,5^.

#### Reagents & chemicals

Propamidine isethionate (PI) powder was a kind gift from The Egyptian Orchidia Pharmaceutical Company. Chitosan (CS), with a molecular weight of 200 kDa and a grade of acetylation above 90%, came from Alpha-Chemika, India. The source of sodium tripolyphosphate (TPP) was from Indian Loba-Chemie. The kind donation of Lipoid S100 (LS100) (soybean-phosphatidylcholine) came from Lipoid GmbH, located in Germany. The supplier of cholesterol (CHOL) was the United Kingdom’s Sigma Chemicals Corporation. We bought hydroxypropyl methylcellulose (HPMC) from Alpha Sort in the United States, with a viscosity of 4000 cp. Poloxemar 407 (Pluronic F-127), Poloxamer P 188 (P 188), and stearic acid (SA) were given as gift samples from Pharco Pharmaceutical Industry, Alexandria, Egypt. The source of castor oil was Morgan Chemicals in Cairo, Egypt. Dialysis bag made of cellulose and acetate, VISKING (Serva Electrophoresis, Germany) having a 12,000–14,000 MW cut-off was used. BROLENE Eye drop was purchased from local pharmacies on the Egyptian manufactured by Sanofi- Aventis Co. and used for comparison. Any other substance, including solvents, was of analytical quality.

### Methods

#### Preparation of propamidine isethionate-loaded chitosan nanoparticles (PI-CS NPs_disp_)

Ionic gelation of CS by TPP was used to prepare propamidine isethionate-loaded Chitosan NPs (PI CS-NPs), by the previously described methodology^54–57^. Chitosan was dissolved in 1% v/v acetic acid solution at ambient temperature and stirred for twenty four hours to get 2 mg/ml chitosan solution. 5 N NaOH was used to adjust the pH to 4.5, and then a 0.45 μm filter was used for filtration. The 10 mg of PI was dissolved in 5 ml of chitosan solution by first dissolving it in the least amount of ethanol. The solution was agitated for 24 h at ambient temperature and 300 rpm using a magnetic stirrer to create the CS-PI complex. To stop the particles from aggregating, 0.5 millilitre of 0.05% (Tween 80) was added after 24 h. TPP was dissolved at a concentration of 0.5 mg/ml in purified water with no ions (deionized) for the ionic gelation and cross-linking process. By mixing 5 ml of TPP solution by dropping with 5 ml of chitosan solution (2 mg/ml, Table 1), PI dispersions (PI-CS NP_disp_) were produced. After stirring the mixture for 30 min at ambient temperature and 1200 rpm, it was left overnight.

**Table 1:** The composition of Propamidine isethionate (PI) loaded nano-dispersion formulations.

Formulation code	CS ^a^ (mg/ml)	TPP ^a^ (mg/ml)	Tween 80 (%v/v)	SA (%w/v)	Castor oil (%w/v)	P 188 (%w/v)	LS100 (%w/v)	CHOL (%w/v)
PI-CS NPs	2	0.5	0.1	–	–	–	–	–
PI-NLC	–	–	–	3	1	0.05	–	–
PI-LPs	–	–	–	–	–	–	4	1

All formulations contain PI (0.1% w/v) each in 10 ml volume. ^a^ ;(CS solution : TPP solution = 1:1). CS, chitosan; TPP, tripolyphosphate; SA, Stearic acid; P 188, Poloxamer P 188; LS100, Lipoid S 100; CHOL, Cholesterol.

#### Preparation of propamidine isethionate-loaded nanostructured lipid carrier nanoparticles (PI-NLC_disp_)

Propamidine isethionate (0.1% w/v) and (4% w/w) of lipid phase were used to create NLCs utilizing a modified high-shear homogenization technique^58^. Castor oil was used as the liquid lipid and stearic acid as the solid lipid in a 1:3 ratio. The lipid phase was heated at 75 ± 0.5 °C. The propamidine isethionate was incorporated and stirred with the melted fats using magnetic stirrer (Snijders, Germany). The, Poloxamer (P 188) aqueous emulsifier solution (0.05% w/v) was heated to almost the same temperature of lipids at the same time and then added to the heated lipid phase. It was then homogenized for five minutes at 20,000 rpm using homogenizer (Ultra Turrax T25, IKA, Germany). The heated nanoemulsion was created, permitted to cool to ambient temperature, and after that allowed to refrigerate at 4 °Cto obtain NLCs^58^.

#### Preparation of propamidine isethionate-loaded liposomes (PI-LPs_disp_)

To create PI-LPs, a modified Thin-Film-Hydration Technology was employed^59,60^. Briefly, the lipid phase consisted of 200 mg lipoid S 100 (LS100 = 4% w/v) and 50 mg cholesterol (1% w/v) (based on final liposome (LP) dispersion) were dissolved in 4 ml chloroform. Following that, the above-mentioned solution was mixed with a known amount of pentamidine isethionate (PI) (0.1% w/v). To form a thin layer of lipid on the flask wall, the organic solvents were subsequently removed under low pressure in a rotating evaporator (Rotavapor, Büchi, Germany) around 60 °C. Once the dry residue started to form, the evaporation was run for a further two hours to remove all of the solvent. To generate a suspension, the dried lipid film was hydrated for one hour at 60 °C using 5 mL of phosphate buffer solution (STF) at pH 7.4. PI-LPs were finally produced by sonicating the aforementioned vesicle dispersion for 3 min at 50 °C in a water bath, followed by 10 min sonication at ambient temperature. The resultant vesicle dispersion was put through extrusion cycles through membrane filters with decreasing pore sizes (once through 0.45 mm filter and twice through 0.20 mm filter) and sonication in an ice bath (30 min intermittent)^61^. The dispersion vesicles were refrigerated at 4 °C, sealed, and shielded from light.

### Preparation of Chitosan/Pluronic pH- thermosensitive in-situ gel

The cold method reported by Schmolka was used to create in-situ gels based on Pluronic F-127^62^. The pH-thermosensitive transparent sols were prepared using the estimated concentrations of 20% Pluronic F-127 and 0.5% of chitosan for further characterization^27^. In summary, a solution of 1% v/v acetic acid had been stirred overnight after chitosan (CS), 0.5%, was dispersed it. In chitosan solution, Pluronic F-127 (20% w/v) was dissolved, mixed for three hours, and then refrigerated for the entire night. The PI drug was dispersed in the cold in-situ gel for PI-containing in-situ gels (PI gel), producing a transparent solution.

### Preparation of PI nanoparticles (PI NPs) loaded in-situ gel

After preparing the three distinct nanoparticle procedures previously, the NPs were introduced to a clear, homogenous solution containing 0.5% chitosan CS and 20% F-127, where they were distributed^27^.

#### Preparation of PI-CS NPs in-situ gels

For PI-CS NPs in-situ gel preparation, the dispersion of CS NPs was supplemented with 1% v/v glacial acetic acid. Overnight, chitosan powder (0.5%) was mixed throughout the PI-CS NPs _disp_. In the PI-CS NPs-chitosan mixture, F-127 was added. A final PI concentration of 0.1% w/v was developed in the final in-situ gel formulation.

#### Preparation of PI-NLC in-situ gels

For PI-NLC NPs in-situ gel preparation, the dispersion of NLC NPs was supplemented with 1% v/v glacial acetic acid. The acidified PI-NLC NPs were mixed with 0.5% w/v chitosan powder and left overnight to stir. In the PI- NLC-chitosan mixture, F-127 was added. A final PI concentration of 0.1% w/v was developed in the final in-situ gel formulation.

#### Preparation of PI-LPs in-situ gels

For PI-LPs NPs in-situ gel preparation, the dispersion of LPs NPs was supplemented with 1% v/v glacial acetic acid. The acidified PI-LPs NPs were mixed with 0.5% w/v chitosan powder and left overnight to stir. In the PI-LPs NPs-chitosan mixture, F-127 was added. A final PI concentration of 0.1% w/v was developed in the final in-situ gel formulation.

### Characterization of an PI ocular nano-formulations

#### Colloidal characterization of PI-loaded (Chitosan NPs, NLC, Liposome) as dispersion

There were three distinct formulations observed for several parameters, such as morphology, size distribution, zeta potential, particle size, and entrapment efficiency. Zetasizer-Nano ZS (Malvern, UK) was used to characterize the distribution (polydispersity index (PDI)), zeta-potential (ZS) and particle size (PS) of three formulations. Following the completion of each measurement in triplicate, the means and standard deviations (SD) were estimated.

##### Percent entrapment efficiency (%EE)

When measuring free (un-entrapped) PI that was isolated from these two colloidal dispersions (PI LP and PI NLC), the percentage EE was calculated using an ultrafiltration/centrifugation method that ran for 30 min at 6000 rpm & 25 °C (Vivaspin 6, molecular weight cut-off 100,000). On the other hand, PI CS NP only required centrifugation at 14,000 rpm for 30 min at 4 °C to isolate the PI and calculate the percentage of EE. Three colloidal dispersions’ NPs had settled, and their supernatant contained free drug. Utilizing the UV-1800 Shimadzu spectrophotometer (Japan), the supernatant for PI at λ max = 260 nm was analyzed (Supplementary Fig. 1). The experiment was carried out three times with the specified mean values. The NPs’ percentage entrapment efficiency (EE%) was calculated using the following equations:

1 $${\text{EE}}\% = (({{\text{Total}}\;{\text{PI}}\;{\text{added}} - {\text{Free}}\;{\text{PI}}})/{\text{Total}}\;{\text{PI}}\;{\text{added}}) \times {1}00\\$$

where the total PI represents the whole drug concentration added to the system, and the free PI represents the concentration of free drug (within the supernatant).

##### Morphology

Using transmission electron microscopy (TEM; 1400 PLUS, Joel Ltd., Japan), the drug’s entrapment inside nanoparticles (NPs), the size and internal morphology of the resulting PI-CS NPs, PI-NLC, and PI-LPs were observed. After that, samples were examined and photos were taken.

##### Stability testing

The three formulations’ dispersions (PI-CS NPs, PI-NLC, and PI-LPs) were stored cold for 3 months at 4 °C. After 3 months, ZP, PdI, and particle size were evaluated. Additionally, variations in EE% as an indicator of drug leakage were observed^27,63^.

#### Characterization of propamidine isethionate (PI) loaded pH-Thermosensitive in-situ gels

##### pH and clarity

The pH of Formulations’ hydrogels before adding (1M NaOH base) was measured using standard pH 7 buffers at ambient temperature, a sensitive microprocessor pH meter was used to measure the pH. The device electrode was directly submerged in the sample, which was placed in glass tubes, to perform the measurements. Before and after gelation, the final appearance of the gel formulations, including their color and clarity, was visually assessed^63,64^. Three replicate readings were taken, and the average was calculated.

##### Gelling pH, temperature and time

Samples of in-situ gel formulations were placed in a beaker, and 1M NaOH was gradually added while being stirred continuously. Viscosity was measured and the pH was examined using a sensitive microprocessor pH meter. The formulation’s temperature was progressively elevated by heating the container in a water bath to determine the gelation temperature. At every temperature, the viscosity change was reported. The temperature & pH at which an abrupt change in viscosity was noticed were identified as the gelation temperature and pH, respectively^64^. It was noted what temperature of the test solution became gel-like. A hot plate kept at 35 °C was used to hold an aluminium pan while 100 μL samples were used to calculate the sol–gel transition time. After that, the pan was tilted 90 degrees, and the number of seconds that the sol stopped flowing was noted^63,65^. Three measurements of the gelation pH, temperature, and time were taken.

##### Rheological studies (viscosity)

Using a rotating viscometer (Brookfield, USA) connected to an S-15 spindle running at 10 rpm for 60 s, the viscosity of sols for both drug-only loaded gel and drug NP-loaded gel was assessed at 25 °C and 37 °C^63^. The formulations’ viscosities were assessed using two temperatures: 25 ± 1 °C and 37 ± 1 °C. In addition to increasing the temperature, 1M sodium hydroxide solution was added to the formulation to raise the pH to 7.4. This allowed gelation to occur^64^.

##### Osmolarity study

The osmolarity was determined using a Knauer Semi-Micro Osmometer K-7400S, which is capable of measuring osmolarity in the case of diluted gels.

##### In-vitro release study

The release of PI formulations was assessed in-vitro using the dialysis-bag diffusion method. The rates of PI release *in-vitro* from PI, PI-LPs, PI-NLC and PI-CS NPs loaded in-situ gels were used to compare with PI eye drops from marketed product (BROLENE 0.1% E.dp). To simulate tear fluid (STF) conditions, the dissolution media is composed of phosphate buffer saline (PBS) at a pH of 7.4^58^. A pre-moistened cellulose-acetate dialysis bag containing 0.5 mg of PI was filled with the test formula, which closed both ends.

The dialysis bag was submerged in 25 ml of PBS-pH 7.4 dissolving media, which was placed in the glass beaker that served as the receptor compartment. A thermostat-equipped horizontal water bath shaker was used to maintain a temperature of 35 ± 0.5 °C while rotating at 50 revolutions per minute (rpm)^63^. At predefined intervals (0.5, 1, 2, 4, 6, 12, and 24 h), a 2 ml sample of the receiving medium was taken and substituted with a 2 ml volume of fresh medium to maintain sink condition. The samples were spectrophotometrically analyzed for PI drug concentration at a λmax of 260 nm. Following the PI calibration curve that was created in a dissolving media (with a concentration range of 1–15 µg/ml), the linear regression equations were used to analyze the data. Three duplicates of each measurement were taken.

### *In-vitro* antiprotozoal- evaluation of the effect of different PI formulations on *Acanthamoeba keratitis* Trophozoite

In sterile 96-well plates, the trials were completed. Each well received 100 μl of the calibrated trophozoite suspension, separately, and 100 μl of PI E.dps, PI gel, PI LP NPs gel, PI NLC gel, PI CS NPs Gel were added. Then the plates were sealed and incubated at 37 °C in a 5% CO_2_ atmosphere for 24 h. Parasite control containing only the parasites in PBS (Phosphate Buffer Saline, PH = 7.4) were submitted to the same procedure used for the experimental cultures. Tests were repeated 3 times. After incubation, 100 μl from each well was transferred into 100 μl of 0.3% basic methylene blue media left for 10 min and then counted by the hemocytometer^66^.

2 $${\text{The}}\;{\text{percent}}\;{\text{of}}\;{\text{growth}}\;{\text{reduction}} = \left( {{\text{X}} - {\text{Y}}/{\text{X}}} \right) \times 100$$

where Y is the mean number of stained viable amoebae in cultures treated with PI E.dps, PI gel, PI LP NPs gel, PI NLC gel, or PI CS NPs Gel, and X is the mean number of stained viable amoebae in control cultures^66^.

### Statistical analysis

Each in vitro test was run three times, and the results were given as the mean ± SD. ANOVA was employed to statistically assess the data. The threshold for statistical significance was set at a difference with a *p*-value of ≤ 0.05. The Student–Newman–Keuls multiple comparison test was performed after a one-way ANOVA to compare the various experimental groups. The *p*-value of less than or equal 0.05 was used to determine the significance level of the results. All statistical evaluations were performed using Graph-Pad Prism, version 6 software.

#### Investigated correlation

Investigating the relationship between in vitro pharmaceutical and in vitro antiprotozoal (parasitological) processes involved plotting the mean percent D.E. (dissolution efficiency) against percent inhibition of *Acanthamoeba*. A linear regression analysis was conducted to estimate the robustness of the correlation and evaluate its strength and significance. The *p*-value and R^2^ were calculated^55,67,68^.

## Results and discussion

### Morphology and other DLS parameters

**Fig. 1:**
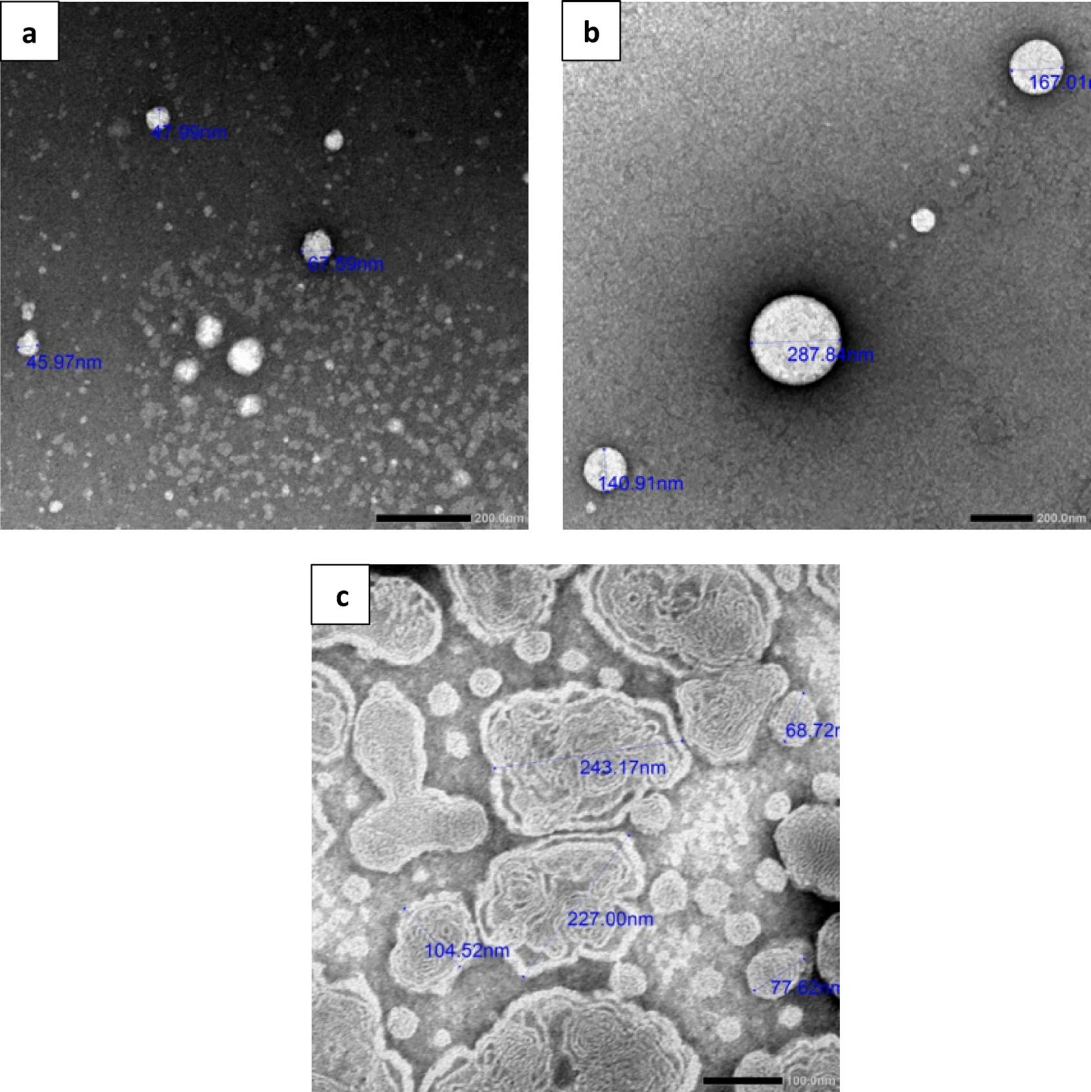
TEM photographs of (**a**); PI-loaded Cs-NP nanoparticles and (**b**); PI-loaded NLC nanoparticles (**c**); PI-loaded liposome nanoparticles.

The TEM pictures of the PI-CS NPs, PI-NLC, and PI-LPs (Fig. 1) demonstrated that the NPs formed at a small size with a spherical form in case of CS Nps and NLC, however in the case of liposomes, they are spherical, heterogeneous, and have a double-layer structure, indicating that they are unilamellar vesicles^69^. Because human eyes can only tolerate particles smaller than 10 um, it is important to note that these spherical nanoparticles have more effective patient compliance and can cross the biological barrier (cornea)^70^. The drug’s entrapment inside the nanoparticles (CS NP, Fig. 1a), (NLC, Fig. 1b), and (LP, Fig. 1c) can be seen in Fig. 1.

Preparing formulas with small particle size (PS) was one of the study’s primary objectives. Because, the smaller particles could penetrate the corneal mucosal membranes more deeply. The results (Table 2) showed that the PS of the studied three nanosystem formulae might be arranged as follows in ascending order: PI-CS NPs, PI-LPs and PI-NLC for both freshly prepared formulaes and after 3 months storage at 4 °C.

**Table 2:** Physicochemical characteristics of Propamidine isethionate (PI) loaded different nanoparticles dispersion formulations before and after 3 months storage at 4 °C.

Formulation code	PI-CS NPs _disp_		PI-NLC _disp_		PI-LPs _disp_	
Time	Freshly prepared	After 3 months	Freshly prepared	After 3 months	Freshly prepared	After 3 months
Visual observation*	√	√	√	√	√	√
Particle size ± SD (nm)	171.6 ± 4.65	190.5 ± 1.50	365.0 ± 40.7	410.3 ± 4.51	199.2 ± 5.28	242.7 ± 8.74
PDI ± SD	0.291 ± 0.02	0.295 ± 0.01	0.444 ± 0.02	0.485 ± 0.02	0.312 ± 0.01	0.355 ± 0.02
Zeta potential (mV ± SD)	+ 15.4 ± 0.93	+ 15.1 ± 0.25	− 32.8 ± 1.06	− 34.5 ± 1.12	+ 7.37 ± 1.64	+ 6.65 ± 0.65
%EE ± SD	77.25 ± 2.08	75.60 ± 0.42	49.82 ± 7.99	44.80 ± 3.60	94.36 ± 1.20	90.40 ± 0.60

All formulations contain PI (0.1% w/v) each in 10 ml volume.

PDI, polydispersity index; %EE, % Entrapment Efficiency.

*Opalescent colloidal dispersion (√).

Furthermore, the estimated PDI values are shown in (Table 2). All of the three PI formulations demonstrated PDI values in the (0.291–0.444) range and (0.295–0.485) range for both freshly prepared formulations and after 3 months storage at 4 °C respectively, which is an acceptable midrange^71^.

Zeta potential is an important parameter to evaluate the stability of particles within formula as well as mono-dispersity^72^. High zeta potential (negative or positive) indicates that there is a strong enough double layer repulsion effect between the particles, which prevents their aggregation^73^. The values of ZP listed in Table 2 ranged between + 7.37 and − 32.8 mV. The generated nanodispersion could not aggregate due to the significant repulsion forces provided by the high ZP^56,74^ especially in case of PI-CS NPs_disp_ (+ 15.4) and PI-NLC_disp_ (− 32.8). Also, zeta potential can predict interactions with surfaces such as with mucin corneal surface. The high (+ ve) charge of (PI-CS NPs) will show the best one, due to the presence of free amino groups of chitosan that give it a positive charge, which it may utilize for interaction with the negatively (−ve) charged corneal surface mucin.

All three nanosystem formulas had %EE above 75% except in case of PI-NLC_disp_ (Table 2). These findings demonstrated the potential for successfully entrapping the PI drug inside the chitosan and liposome nanoparticles. The results (Table 2) showed that the %EE of the studied three nanosystem formulae might be arranged as follows in ascending order: PI-NLC, PI-CS NPs and PI-LPs for both freshly prepared formulaes and after 3 months storage at 4 °C.

According to previous results, the optimum nanoparticle formulation for PI NPs loaded in-situ gel was PI CS NPs with a minimal monodispersity and particle size (PS and PdI, 171.6 ± 4.65 nm and 0.291 ± 0.02, respectively). PI- CS NPs_disp_ possessed a moderately positive ZP (+ 15.4 ± 0.93 mV). PI CS-NPs_disp_ showed a relatively high EE% of + 77.25 ± 2.08%**.**

Mean particle size of PI NLC_disp_ was 365.0 ± 40.7 nm, PdI 0.444 ± 0.02, and zeta potential − 32.8 ± 1.06 mV; the most likely cause of the negative (-ve) charge is the anionic nature of the lipids^75^. PI NLC_disp_ showed a relatively low EE% of 49.82 ± 7.99%.

Concerning PI LP_disp_ , the mean particle size of was 199.2 ± 5.28 nm, PdI 0.312 ± 0.01, and zeta potential + 7.37 ± 1.64 mV; compared to neutral and negatively charged liposomes, these positively charged liposomes exhibit superior corneal penetration^49^. PI LP_disp_ showed high EE% of 94.36 ± 1.20, this may be the main cause of the low dissolution rate of drug.

### Stability storage data

The three PI nanoparticles formulations were stable during the stability testing period (Table 2)**.** After 3 months of being stored at 4 °C, PI nanoparticles demonstrated no significant change in PdI (*p* > 0.05) or particle size, suggesting that the three formulations that were chosen were stable (Table 2). Drug leakage was indicated by measuring the EE%. As demonstrated by Table 2, the EE% of all three PI nanoparticle formulations decreased slightly (*p* > 0.05) after storage (77.25 ± 2.08, 49.82 ± 7.99, and 94.36 ± 1.20 at zero time compared to 75.60 ± 0.42, 44.80 ± 3.60, and 90.40 ± 0.60 after 3 months for PI CS-NPs, PI NLC, and PI-LPs, respectively).

### Preparation and characterization of different PI- NPs formulas loaded pH-Thermosesitive in-situ gel

Ocular in-situ gels are a promising alternative to overcome the drawbacks of conventional eye drops as they link the advantages of solutions such as accuracy & reproducibility of dosing, or ease of administration with extended contact time^35^. The development of a pH-thermosensitive gel that is sol (liquid) at ambient temperature to facilitate easier instillation and quickly solidifies into gel upon contact to physiological tears pH of 7.4 and precorneal temperature of 35 °C for extended ocular retention was one of the main goals of this work^63,64^. A mixture of chitosan, a pH-sensitive polymer that also enhances permeability, and pluronic F-127, a thermosensitive polymer, was utilized as a gelling agent^64^.

Also, the constituents of in situ gel are eco-friendly from natural materials such as chitosan which is a natural polymer suitable for use in occular formulations due to its safe, biocompatibility, biodegradability, mucoadhesive character, antimicrobial properties, permeation enhancer, and corneal wound healing effects. The combination of chitosan, a pH-sensitive polymer, with other stimuli-responsive polymers such as pluronic F-127, a thermosensitive polymer leads to increased mechanical strength of formulations and an improved therapeutic effect due to prolonged ocular contact time. It helps in the treatment of eye contamination due to its anti-microbial effect and can treat dryness and redness of the eye due to its anti-inflammatory effect. No need to use preservatives like the Marketed eye drop product due to the presence of chitosan in the gel dosage form^35^.

To improve absorption and extend the gel’s duration, PI nanoparticle formulas were mixed into an in-situ gel. 20% w/v of pluronic F-127 was utilized for thermosensitive in-situ gel production; the gel phase formed when the concentration exceeded the micellar concentration^27^.

#### Clarity and pH data

For ocular formulations to prevent patients from feeling discomfort and clouded vision due to non-transparent formulations, clarity is a very helpful feature^63,65^. In both liquid & gel states, all formulations were determined to be transparent, clear, and homogenous; however, PI LP and PI NLC had a milky appearance. Despite the slightly turbid PI-CS NPs dispersion, the PI-CS NPs gel sample was clear and transparent when viewed in the light, and an examination with the eyes revealed no foreign matter, indicating that the gel’s clarity satisfied ophthalmic gel standards.

The pH of each in-situ gel formulation has to be determined to confirm that it is appropriate for use in ocular applications. Before adding 1M NaOH, the pH values of the in-situ gel formulations ranged from 6.2 to 6.8, as seen in Table 3. This is considered to be non-irritating since prior research has indicated that pH values between 4–8 are suitable for ocular application^76^. This environment is acidic, which stimulates the growth of fibroblasts while suppressing the proliferation of microorganisms^77^. Additionally, it has been claimed that the electrokinetic properties of colloids can be determined by the pH^78^. While all formulations’ gelling pH ranged from 6.8 to 7 after adding 1M NaOH, confirming the chitosan polymer’s pH-sensitivity & the gelling action of all PI in-situ gel formulations upon contact with physiological ocular pH of 7.4.

**Table 3:** Physical parameters, gelling properties, viscosity and osmolarity data characterized of P407/CS in situ gels and for PI loaded in situ gel and different NPs-loaded in situ gel (Data are presented as mean ± SD, n = 3).

Formula name	F-127 (%)	CS (%)	Clarity	pH*	Gelling pH**	Gelling Temperature (°C)	Gelling Time (Seconds)	Viscosity at 25 °C (cP)	Viscosity at 37 °C (cP)	Osmolarity (mosmol/kg)
Blank Gel _F-127_	20	–	Clear	6.8 ± 0.1	6.8 ± 0.1	45.5 ± 0.50	> 120	15.5 ± 2.3	680 ± 24.9	202 ± 3.5
Blank Gel _F-127/CS_	20	0.5	Clear	6.6 ± 0.1	6.8 ± 0.2	34.8 ± 0.25	50 ± 2.00	51 ± 8.5	1051 ± 33.1	230 ± 5.5
PI Gel	20	0.5	Clear	6.5 ± 0.2	6.8 ± 0.1	34.2 ± 0.26	30 ± 1.73	83 ± 8.0	1160 ± 53.3	450 ± 1.5
PI LPs Gel	20	0.5	Milky	6.4 ± 0.3	7.0 ± 0.2	33.4 ± 0.17	12 ± 1.00	145 ± 9.0	1532 ± 42.5	190 ± 6.1
PI NLC Gel	20	0.5	Milky	6.4 ± 0.1	6.9 ± 0.1	33.5 ± 0.31	11.3 ± 0.58	138 ± 2.0	1420 ± 26.5	462 ± 2.5
PI CS NPs Gel	20	0.5	Clear	6.2 ± 0.2	6.8 ± 0.1	33.0 ± 0.15	15 ± 2.00	95 ± 5.2	1210 ± 51.6	208 ± 3.1

*pH of formulations before adding 1M NaOH; **pH of formulations after adding 1M NaOH.

#### Gelling pH, temperature and time data

The formulation transforms into gel at the gelation temperature and time when it no longer exhibits fluidity. These factors are essential to ocular delivery to prevent lacrimal fluid draining and to promote successful gel creation even after diluting the tiny amount of tear fluid^63,79^.

A prior study found that the optimal temperature range for ocular administration is 25–37 °C. To avoid issues with formulation administration, manufacturing, and handling, the product’s gelling temperature should be higher than 25 °C. A previous study found that when the concentration of Pluronic F-127 rises, the sol-to-gel transition temperature decreases^27,80^. Mucoadhesive polymers, like chitosan (CS) at a concentration of 0.5%, can be added to the formulation to extend its residence time, which would result in a notable drop in the temperature and gelling time^27^. The pH range of the in-situ gel of PI formulations was 6.2–6.6. The gelation temperature was determined to be 33–34.8 °C, while the gelation pHs ranged from 6.8 to 7.0. The results conclusively showed that when the formulations’ pH and temperature are increased, all four in-situ PI formulations turn into gel exhibiting significant increases in viscosity. The formulations were liquid at ambient temperature and the formulation pH (pH 6.2–6.6), according to the results, they quickly transitioned into the gel phase at the physiological temperature of 37 °C and the pH of the tear fluid (pH 7.4).

In this study, chitosan, a pH-sensitive polymer, served as a permeability enhancer while Pluronic F-127, a thermosensitive polymer, served as a gelling agent. The concentration of Pluronic F-127 was fixed at 20% in order to obtain the appropriate gel transition-temperature in the eye.

The composition of the prepared in-situ hydrogels, together with the temperatures and gelation times, are displayed in Table 3. The gelling temperature and time (at 20% F-127) were significantly reduced upon the addition of 0.5% chitosan to Pluronic F-127. Similar to this, when the pH was raised to 7.4, all three types of PI nanoparticles in the F-127/CS gel (PI-CS NP, PI-NLC, and PI-LP) showed reduced gelling temperature and gelling time as well as increased gel viscosity. A similar observation was reported by Khallaf et al.^63^, Huang et al.^81^ and Almeida et al.^82^, where the addition of liposomes, chitosan nanoparticles or nanostructured lipid carriers to F-127 resulted in a decrease in gelling temperature.

Considering that the central corneal temperature ranges between 32.8 and 35.4 °C^83^, the gelling temperature of four formulas of the PI loaded in-situ gel were between 33–34.2 °C, which were appropriate and within the ophthalmic temperature. As a result, the four PI gel formulae (PI gel, PI CS NPs gel, PI NLC gel, and PI LP gel) would be liquid at ambient temperature and turn into a gel in less than a minute upon ocular administration. The longer duration of contact in the eye may be attributed to the F-127/CS in situ gel’s thermal reversibility.

#### Viscosity data

The PI in-situ gel formulations should have an appropriate viscosity for easy instillation, after which it is converted into the gel. The physiological body temperature (37 °C) and room temperature (25 °C), respectively, were used to measure the viscosity (Table 3). The formulations were liquid and had low viscosities at 25 °C due to their thermosensitivity; as the temperature increased, they became gel and showed higher viscosities.

When Blank Gel _F-127_ (Table 3) was heated from room temperature to physiological temperatures, its viscosity increased noticeably. This could be explained by the fact that the hydrated polypropylene oxide component of the poloxamer (Pluronic) chains suffers dehydration at low temperatures and that this causes chain entanglement when the temperature is raised^27^. In terms of how viscosity changed with temperature, Blank Gel _F-127/CS_ displayed properties similar to pluronic F-127 alone, but with greater total viscosity values because of the chitosan (CS) inclusion. Amino groups may have contributed to the enhanced viscosity in the presence of CS by increasing the number of hydrogen bonds and the gel’s mechanical strength^35^.

A small but significant increase in viscosity (*p* < 0.05) was observed when PI nanoparticles were added to an F-127-based gel. This increase was caused by an increased membrane fluidity as a result of interior thickening, which may have been caused by F-127 molecules interfering with liposome lipid bilayers^84^, lipids of nanostructured lipid carriers (NLC)^82^ and with chitosan nanoparticles^81^.

At 25 °C, the viscosity of the PI, PI-LPs, PI-NLC, and PI-CS NPs gels revealed, respectively, values of 83,145,138, and 94 at 10 rpm. However, it was significantly greater at 37 °C, with values for PI, PI LPs, PI NLC, and PI CS NPs gels equal to 1160, 1530, 1400, and 1210 at 10 rpm, respectively. The behavior of pH-temperature-dependent gelation could be described as follows: Long-lasting coils on pluronic F-127 chains are protected in a hydration layer at 25 °C. Desolvation, on the other hand, results from the H-bond between PPO units and water dissolving at higher temperatures. Viscosity increases as polymer chains get closer to each other and interact more. It should be noted that the gels’ viscosity was influenced by the Pluronic F-127 level and the (CS) polymer that was used.

#### Osmolarity data

The osmolarity data of all formulations are shown in (Table 3). It is more necessary to obtain an isotonic solution for creating ophthalmic formulations. The tonicity limitations in practice can vary from (0.5–5%) NaCL, or around171 mOsm/kg to roughly 1711 mOsm/kg, without causing noticeable eye discomfort^85,86^.

Our formulations meet the specified standards when comparing the results in Table 3 with the particular requirements for ophthalmic formulations, which have an osmolarity between 190 and 462 mosmol/kg. Consequently, these findings suggest that this physicochemical parameter of our formulations shouldn’t cause any discomfort or irritation.

### *In- vitro* release rates for different PI formulations

The in-vitro release patterns of PI loaded in-situ gels were evaluated in phosphate buffer saline (PBS) at pH 7.4 to mimic tear fluids over a 24-h period in comparison to commercially available PI eye drops (Brolene^®^) using the dialysis-bag method.

Comparing various in-situ gel formulations to commercially available PI eye drops, the *in-vitro* release of PI from each formulation was examined to see if the formulations might control the release of the entrapped drug. Consequently, the drug release of the studied formulae might be arranged as follows in ascending order: PI-LP gel, PI-NLC gel, PI-CS NPS gel, PI gel, and PI E.dps. In this case, PI release was affected by the gel matrix & solubility of the drug.

**Fig. 2:**
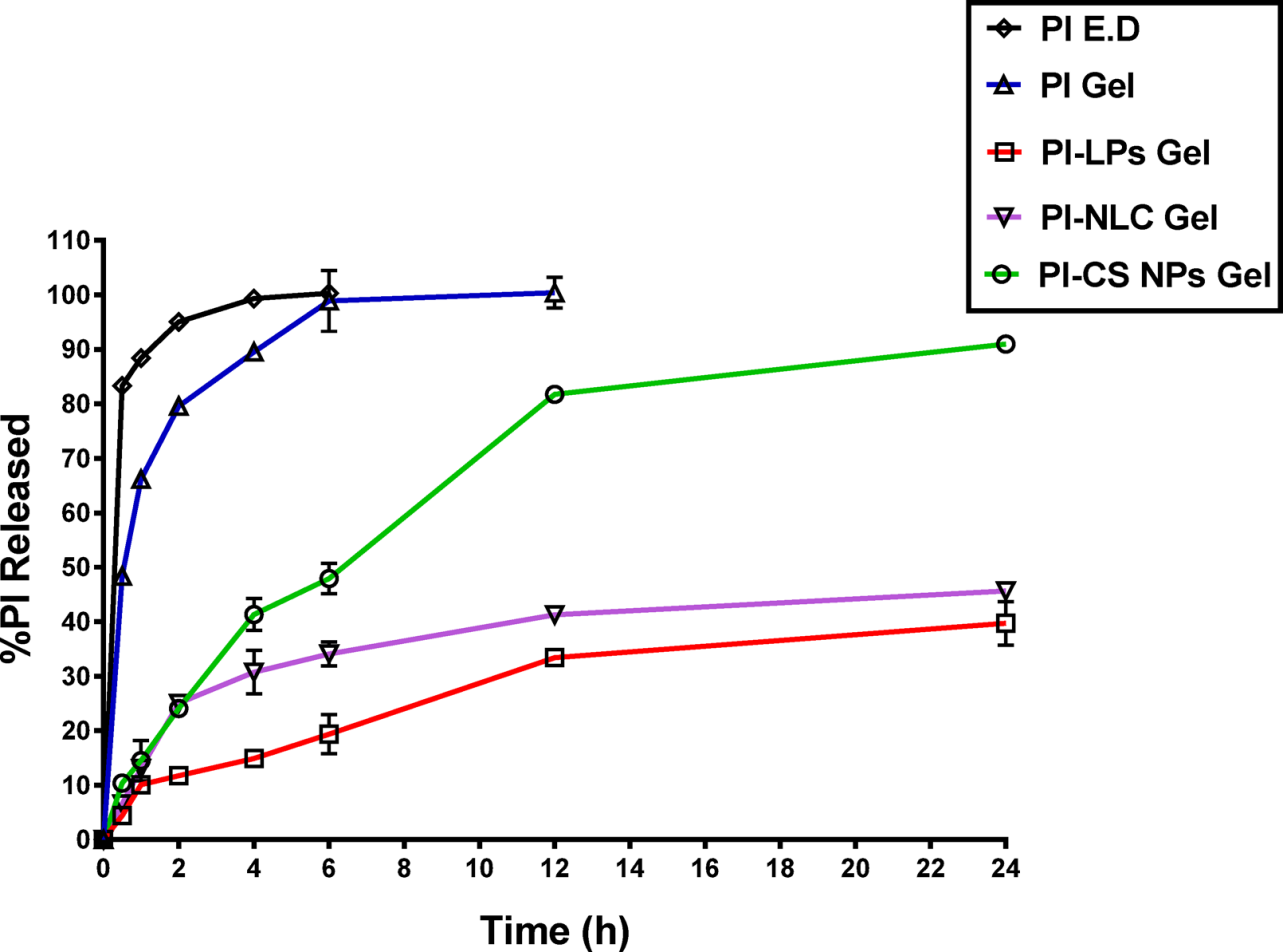
In- vitro release profiles of PI from three NPs formulas based in-situ gel and PI E.D and PI based in-situ gel in dissolution medium (PBS of pH 7.4).

The three gel formulae for PI nanoparticles demonstrated a sustained or prolonged release over 24 h in the *in-vitro* release profiles when compared to commercial eye drops and PI gel (Fig. 2). The three PI nanosystems filled in-situ gels exhibited a gradual slow release of PI, indicating that the drug was uniformly entrapped throughout the systems. Furthermore, the drug release was further reduced as a result of the inclusion of nanoparticulate dispersion into in-situ gel. The biphasic release behavior of the three PI nanoparticle gel formulas (CS, NLC, and Liposome) is also readily observable; these release profiles show a relatively quick release within the first two hours (11.7–25%), which is followed by a more delayed release pattern. One possible explanation for the first burst release from the gel loaded with NPs is that the free drug molecules attached to the NPs surface released quickly, whereas the entrapped drug molecules released more slowly^87^.

The PI-liposome-loaded gel exhibited the lowest drug release profile, indicating that drug diffusion across the lipid bilayer, rather than liposome disruption, was the primary mechanism for release^88^. Theoretically, compared to other PI formulations, liposomal gel should release particles more slowly overall because of the combination of transport resistance caused by the liposome bilayer, high entrapment efficiency (%EE) of 94%, and gel network^89^. This idea was further validated by a kinetic analysis investigation that demonstrated PI release from liposomes followed the Fickian diffusion mechanism; the best-matched kinetic model was the Korsmeyer-Peppas model (n = 0.5)^90^.

Despite the PI liposome gel exhibiting the lowest drug release formula, this is not needed for ocular application over 24 h. The created PI-CS NPs gel was the most acceptable formula based on data from release profiles, as it was the most capable of releasing PI in a controlled way (Fig. 2), with a release percentage of 91% over 24 h. There is a possible hydrogen bonding between the –OH group of PI and –NH group of the chitosan NP polymer. This may have contributed to the significantly slower release of PI-CS NPs gel than PI gel because it took time for the drug to diffuse outside the nanoparticles^91,92^.

#### Dissolution profile comparison:

After performing the in-vitro release test, a mathematical comparison is calculated using the difference factor (*f1*) and similarity factor (*f2*). The FDA guidelines stated that *f1* values up to 15 (0–15) and *f2* values greater than 50 (50–100) ensure sameness or equivalence of test product to the reference product^93^. For each of the four prepared PI in-situ gels, (*f1*) and (*f2*) were computed^94–96^ in comparison to the commercial Eye drop (Table 4). The results of the two parameters (*f1* over 15 (excluding PI gel) and *f2* below 50) showed that, in terms of release profile, none of the in-situ gels were similar to the commercial eye drops.

**Table 4:** Difference factor (*f*1) and Similarity factor (*f*2) and percent Dissolution Efficiency (% D.E) calculated values for tested PI in-situ gel formulas compared to marketed PI Eye drops as a reference product.

Formula code	*f*1 value	*f*2 value	%D.E_24h_
PI E.D	–	–	22.90 ± 0.22
PI Gel	12.60 ^a^	39.72 ^b^	44.55 ± 1.45 ^c#^
PI LPs Gel	79.97 ^b^	7.22 ^b^	28.07 ± 0.29 ^c^^#^^$^
PI NLC Gel	70.56 ^b^	9.93 ^b^	37.24 ± 1.17 ^c^^#^^$^^@^
PI CS NPs Gel	53.38 ^b^	13.84 ^b^	67.01 ± 1.49 ^c#$@∞^

a: similar; b: different; c: Statistically significant at (*p* ≤ 0.05).

Statistical analysis was done using one-way ANOVA followed by Student–Newman–Keuls multiple comparison test. # *p* < 0.05 vs PI E.D, $ *p* < 0.05 vs PI Gel, @ *p* < 0.05 vs PI LPs Gel, ∞ P < 0.05 vs PI NLC Gel.

Statistical analysis was used to compute a second dissolving parameter, percent dissolution efficiency ( %DE_24h_)^97,98^. The computed statistics support the earlier findings; Table 4 displays a significant variation in %DE between all four PI gel formulations and commercial eye drops.

**Fig. 3:**
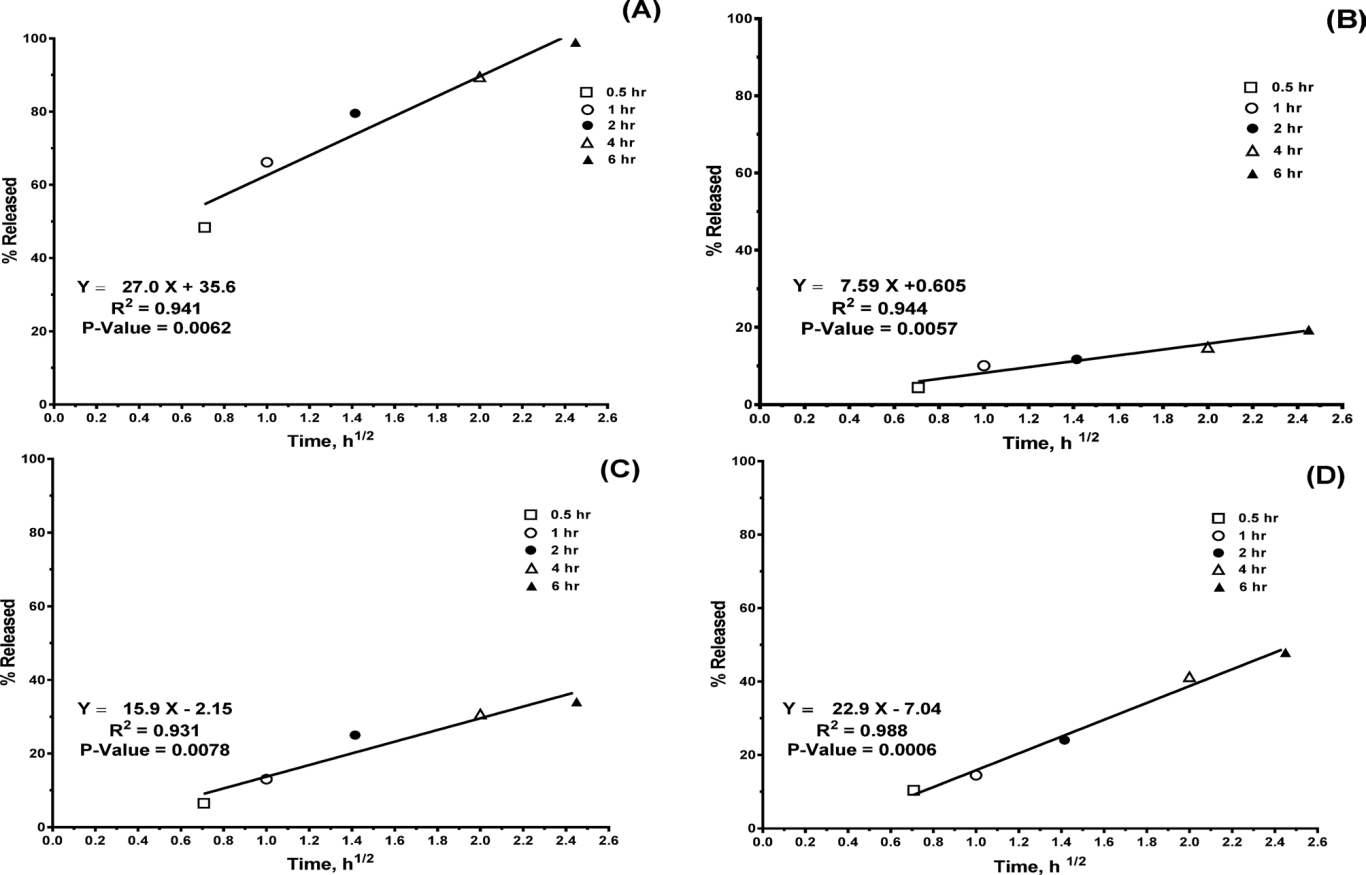
Higuchi release plots; (**a**) PI gel, (**b**) PI NLC gel, (**c**) PI LP gel and (**d**) PI CS NPs gel.

It is essential for studying drug release kinetics and process mechanisms to accurately characterize the drug release or diffusion profile of a delivery system. The drug release data of the PI nanoparticle-loaded gel formulations were fitted with several models, including the Higuchi equation, zero-order, Korsmeyer-Peppas, and first-order models^90,99^. The release patterns of four PI-loaded in-situ gels (PI gel, PI-CS NPs gel, PI-NLC gel, and PI-LP gel) follow Higuchi order kinetics, according to mathematical data modeling. PI eye drops, on the other hand, follow first-order release kinetics (Fig. 3). Higuchi release plots were constructed (Fig. 3)**.** Linearity with high (r^2^) values was observed in the four PI gel profiles suggesting diffusion-controlled release. Korsmeyer et al.’s equation was fitted with the data to confirm the diffusion mechanism. The Korsmeyer-Peppas diffusional exponent (n)^100^, gave values of 0.15, 0.5, 0.34 and 0.5 for PI gel, PI-LP gel, PI NLC gel and PI CS NPs gel respectively with high coefficient r^2^ value between (0.9384–0.9767) indicating Fickian diffusion interpreted as an indication of simultaneous diffusion. The Fickian diffusion-controlled mechanism for PI release from the four studied PI in-situ gels is supported by values of n ≤ 0.5^90,99^.

The extended duration of drug release rates may be related to the elevated viscosity induced by the in-situ gels based on Pluronic F-127^101^. Furthermore, the addition of chitosan (CS) resulted in a retarding effect by raising the total viscosity of the product and distorting or squeezing the extra-micellar aqueous channels of the Pluronic F-127 micelles, which are where the PI diffuses. Therefore, rather than the various nanosystems themselves, the in situ gel acts as an extra barrier to the release of PI.

### Reduction in the number of amoeba treated by different PI formulas compared to parasite control

After 24 h of incubation, the PI chitosan nanoparticles gel demonstrated a 92% reduction in the number of viable amoebae, which was extremely significant when compared to the parasite control followed by PI gel, PI NLC Gel, PI LPs Gel, and commercial PI Eye drops (Table 5).

**Table 5:** Reduction of the number of amoebae in-vitro after 24 h incubation with different Propamidine isetionate (0.1% w/v) formulations in comparison to marketed eye drops.

Code name	The mean number of amoebae ± SD	Inhibition (%)
Parasite control	120.33 ± 1.52	0.00
PI E.D	60.00 ± 1.73	50.1 ± 1.44
PI Gel	24.00 ± 1.63	80.1 ± 1.66^#^
PI LPs Gel	44.33 ± 2.52	63.2 ± 2.09^#^^$^
PI NLC Gel	31.67 ± 1.25	73.7 ± 1.27^#^^$^^@^
PI CS NPs Gel	9.66 ± 1.53	92.0 ± 1.26^#^^$^^@^^∞^

Statistical analysis was done using one-way ANOVA followed by Student–Newman–Keuls multiple comparison test. # *p* < 0.05 vs PI E.D, $ *p* < 0.05 vs PI Gel, @ *p* < 0.05 vs PI LPs Gel, ∞ P < 0.05 vs PI NLC Gel.

The PI-CSNPs in-situ gel formulation offers an effective substitute for traditional BROLENE eye drops in the management of Ocular Acanthamebae, it showed double the effect based on % inhibition, reached 92%. It is a promising alternative to conventional PI eye drops as they carry the advantages of eye solutions such as accuracy & reproducibility of dosing, or ease of administration with prolonged contact time and low frequency.

### Investigated correlation

**Fig. 4:**
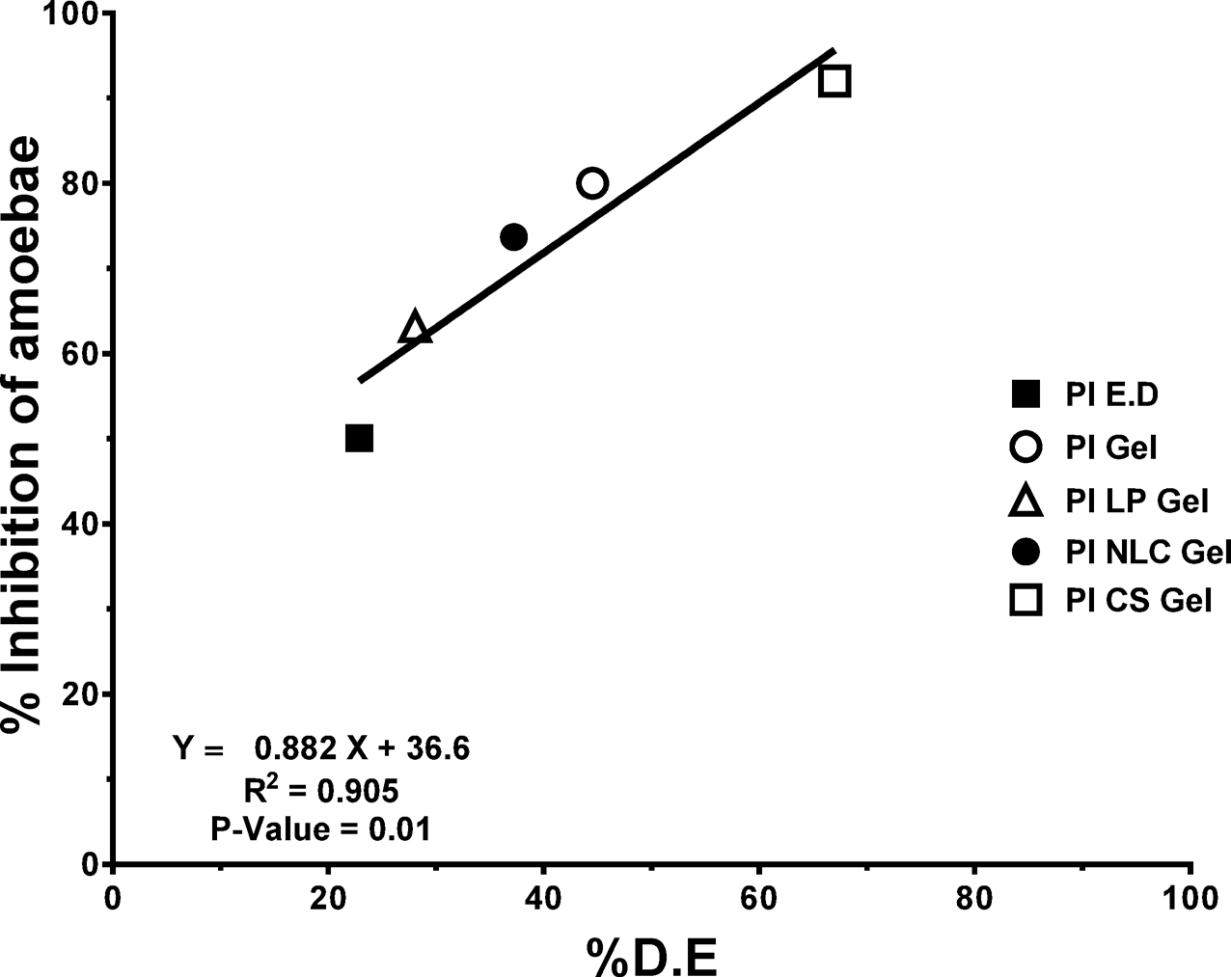
Linear correlation between %D.E against % inhibition of amoebae.

A quantitative relationship between in vitro pharmaceutical data and in vitro microbiological characteristics was investigated. The Dissolution Efficiency, (% D.E) and the % inhibition of *Acanthamoebae* revealed a significant correlation coefficient R^2^ = 0.905 with (*p*-value = 0.01) (Fig. 4).

Lastly, based on the characterization data, all PI nanoparticles in-situ gels generated from the (20% F-127) and (0.5% CS) polymers behaved satisfactorily in terms of in-vitro gelling time & temperature, pH, and viscosity (Tables 3 and 6). Only PI gel and PI-CS NPs gel showed satisfactory concerning clarity, they were transparent homogeneous gels. Table 6 summarizes all the *in-vitro* parameters of PI in-situ gel formulations compared to commercial PI eye drops. The PI CS-NPs in-situ gel was the most acceptable parameters than others, which showing a promising formulation in the treatment of ocular AK.

**Table 6:** Summarization of the in-vitro parameters observed of different PI loaded in-situ gel formulations.

Formula name	Gelling temperature (°C)	Gelling time (Seconds)	Clarity	pH	Viscosity at 25 °C, 37 °C (cP)	Stability	Osmolarity (mosmol/kg)	Sustained in-vitro release (during 24 h)	(%) Inhibition of amoebae
PI Gel	√	√	√	√	√	√	√	x	x
PI LPs Gel	√	√	x	√	√	√	√	x	x
PI NLC Gel	√	√	x	√	√	√	√	x	x
PI CS NPs Gel*	√	√	√	√	√	√	√	√	√

*most accepted formula; √accepted parameter; x: not accepted parameter.

Concerning antimicrobial efficacy outcomes, our in-situ gel formulations, particularly the chitosan/pluronic in-situ gel loaded with PI chitosan-based nanoparticles, offer a significant enhancement in antimicrobial characteristics against AK. As previously noted, studies have shown that chitosan (CS) has antibacterial capabilities through the disruption of microbial membranes^102^. As a result, the chitosan action would work in conjunction with the PI drug to treat the ocular AK infection like an antimicrobial agent.

## Conclusion

Now a days, researchers believe that nano-in-situ-gels hold great promise for delivering drugs to the eyes at the nanoscale. Chitosan/Pluronic F-127 polymers with unique sensitivity to pH and temperature were used in our in-situ gel design of stimulus-responsive effect, which allowed us to improve and regulate PI release from nanogel within ocular pH and temperature conditions. This study describes the creative design of smart hydrogels that contain drugs loaded in various nanocarriers, such as polymeric-based nanoparticles (chitosan NPs) or lipid-based nanocarriers (liposomes, nanostructured lipid carriers), which have been developed for many advantages, including anti-amoebic effect, healing of tissue, and controlled drug release in comparison to marketed eye drop product (Brolene^®^). The results indicated that PI CS-NPs in-situ gel exhibited the greatest outcomes in terms of the in-vitro *anti-aemobic* effect (proved statistically more effective around 92% inhibition which is double the effect compared to the commercial eye drop, based on % amoeba inhibition), exhibiting a longer and regulated drug release, a clear solution, good stability, and the potential to increase bioavailability, which would increase patient compliance than marketed product. In summary, PI chitosan nanoparticles have demonstrated a promising Nano-Ocular Delivery System loaded pH-Thermosensitive in-Situ Gel promoted for effective local treatment against *Acanthamoeba keratitis* with reduced application frequency, and less daily amount of applied PI compared to a commercial eye drop application. This system can serve as a viable substitute for the traditional marketed PI ocular drug delivery system.

## Electronic supplementary material

Below is the link to the electronic supplementary material.

Supplementary Material 1

## Data Availability

Data is provided within the manuscript or supplementary information files.
