# Supplementary material for: Comparative evaluation of nano ocular delivery systems loaded pH and thermosensitive in situ gels for Acanthamoeba keratitis treatment
Source: Sci Rep. 2025 Jun 3;15:19430. doi: 10.1038/s41598-025-03418-5 (PMC12134219; doi:10.1038/s41598-025-03418-5)
Supplement: Supplementary file 1 — Supplementary Material 1 [file 41598_2025_3418_MOESM1_ESM.docx]

**Supplementary Figure 1**

Supplementary Figure 1: Calibration graph of Propamidine isethionate solution using UV spectrophotometry at 260 nm in the range 1-10 µg/ml.
